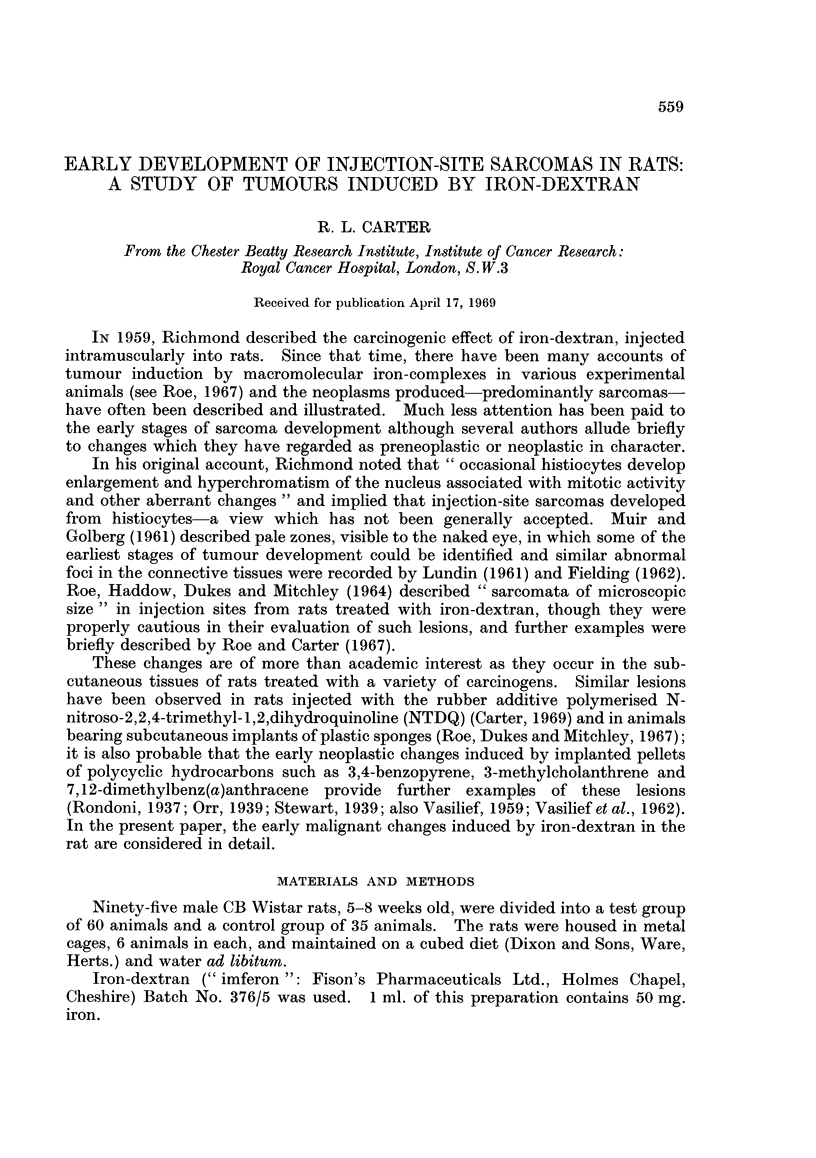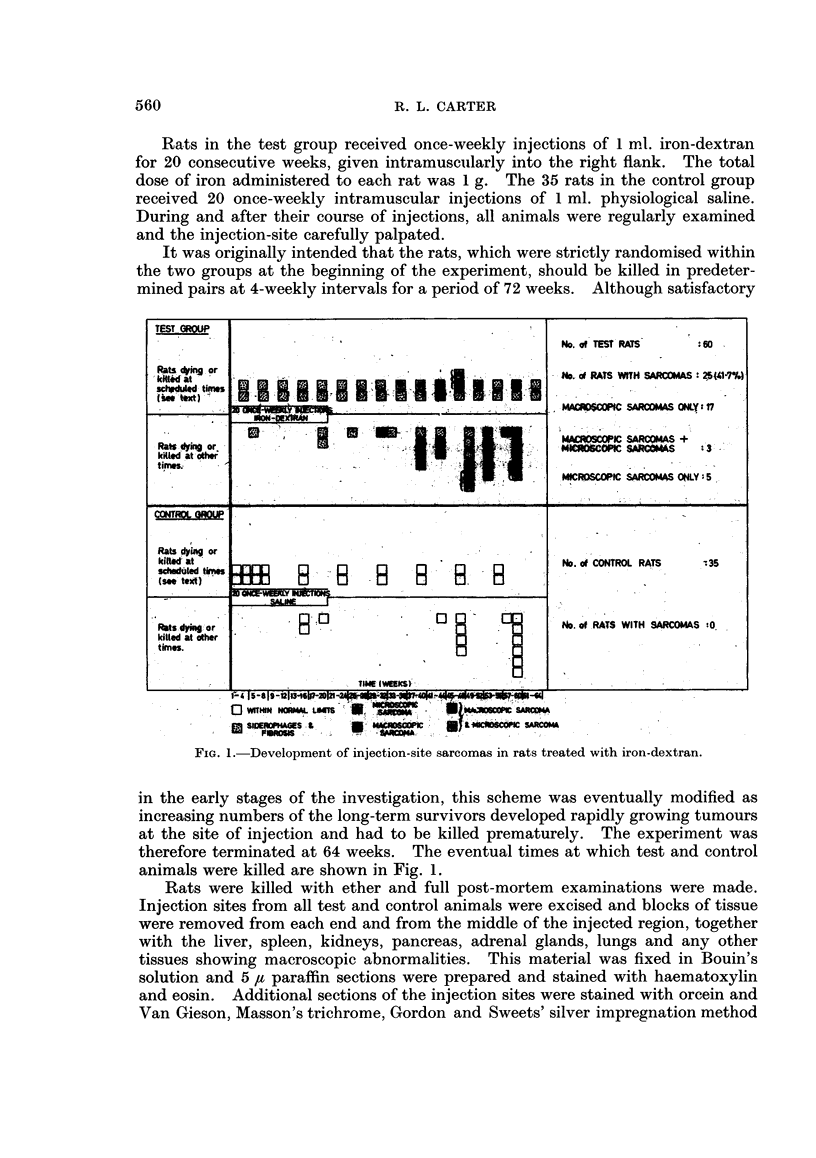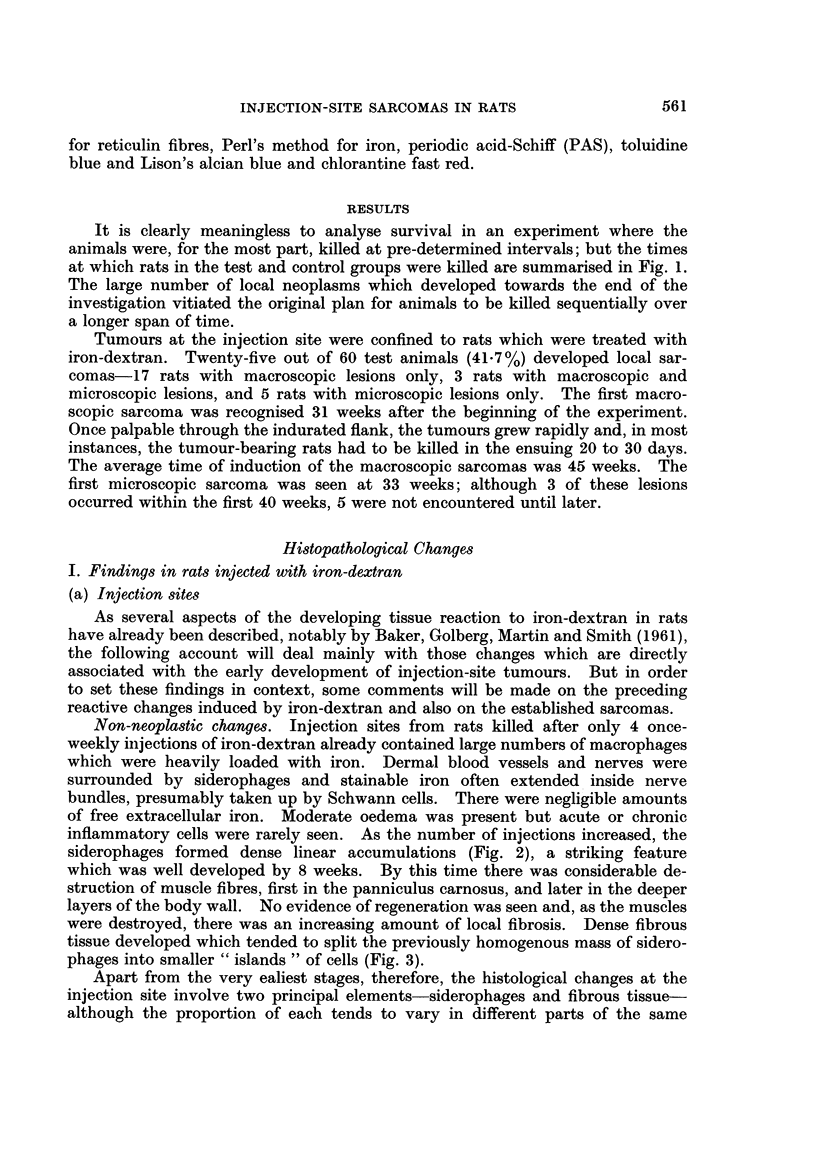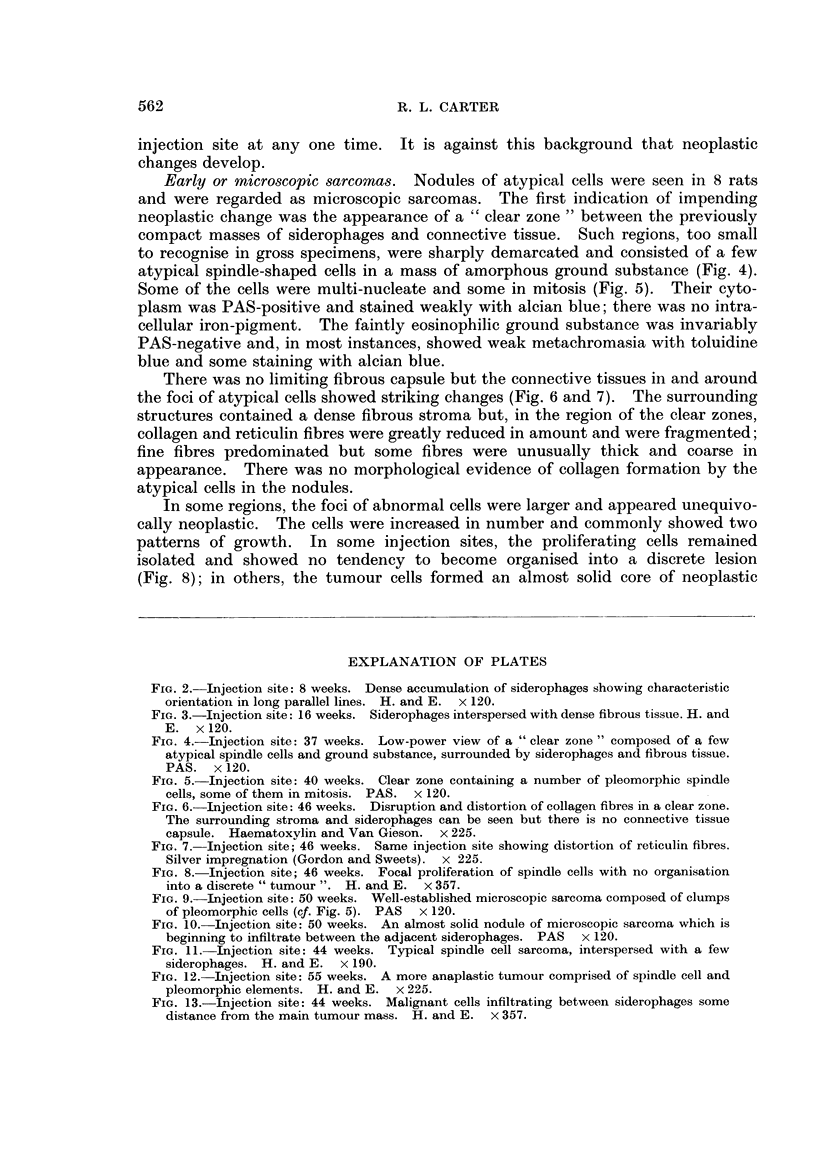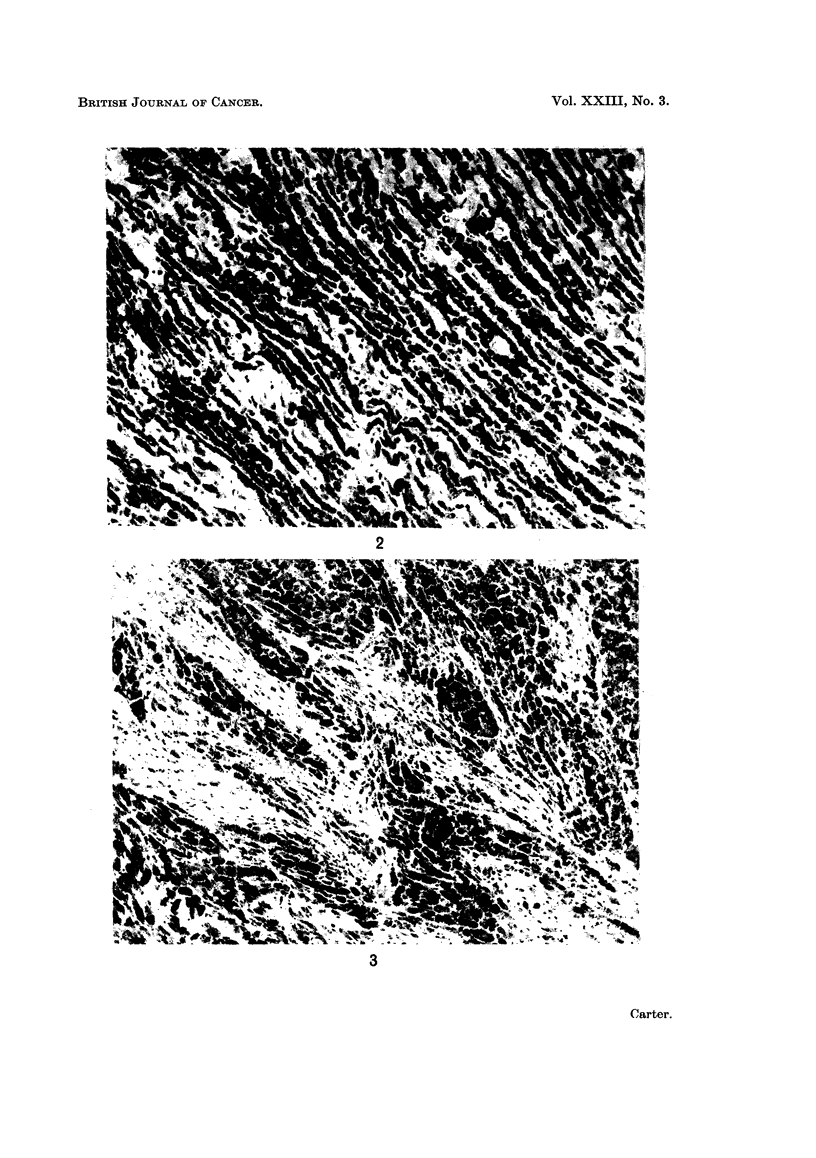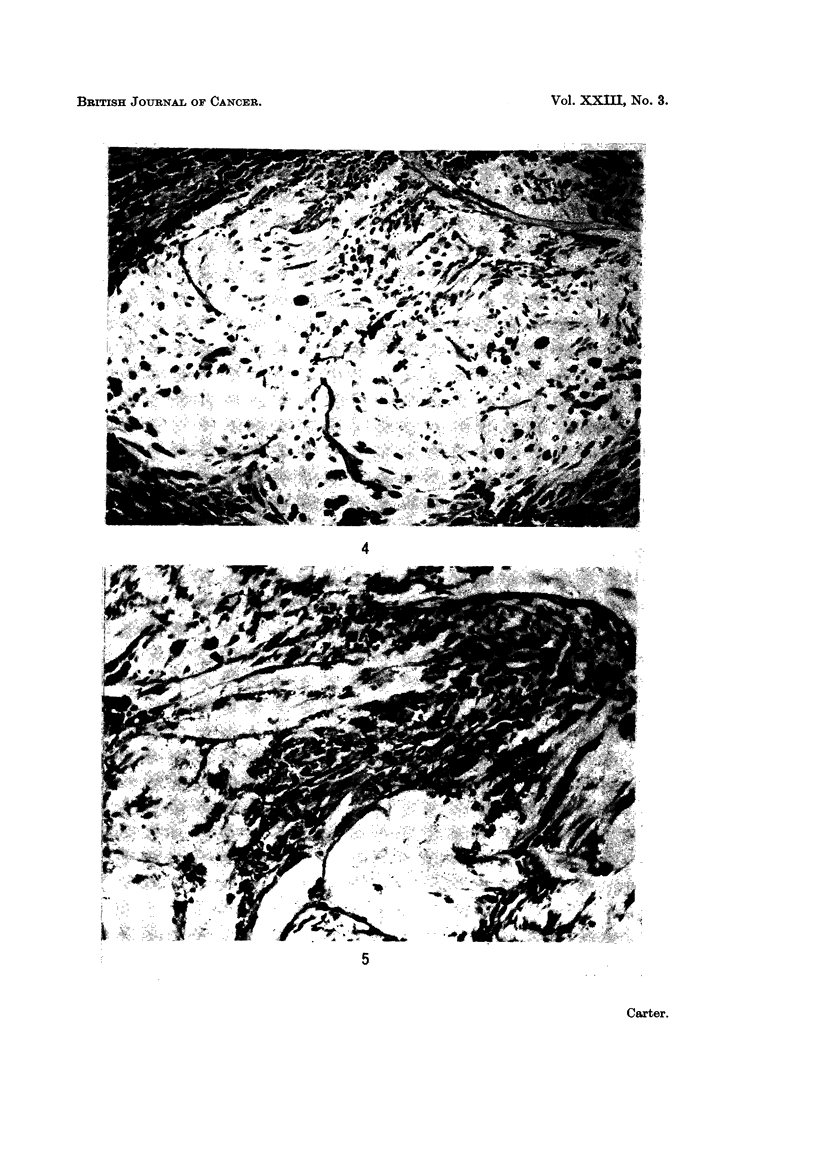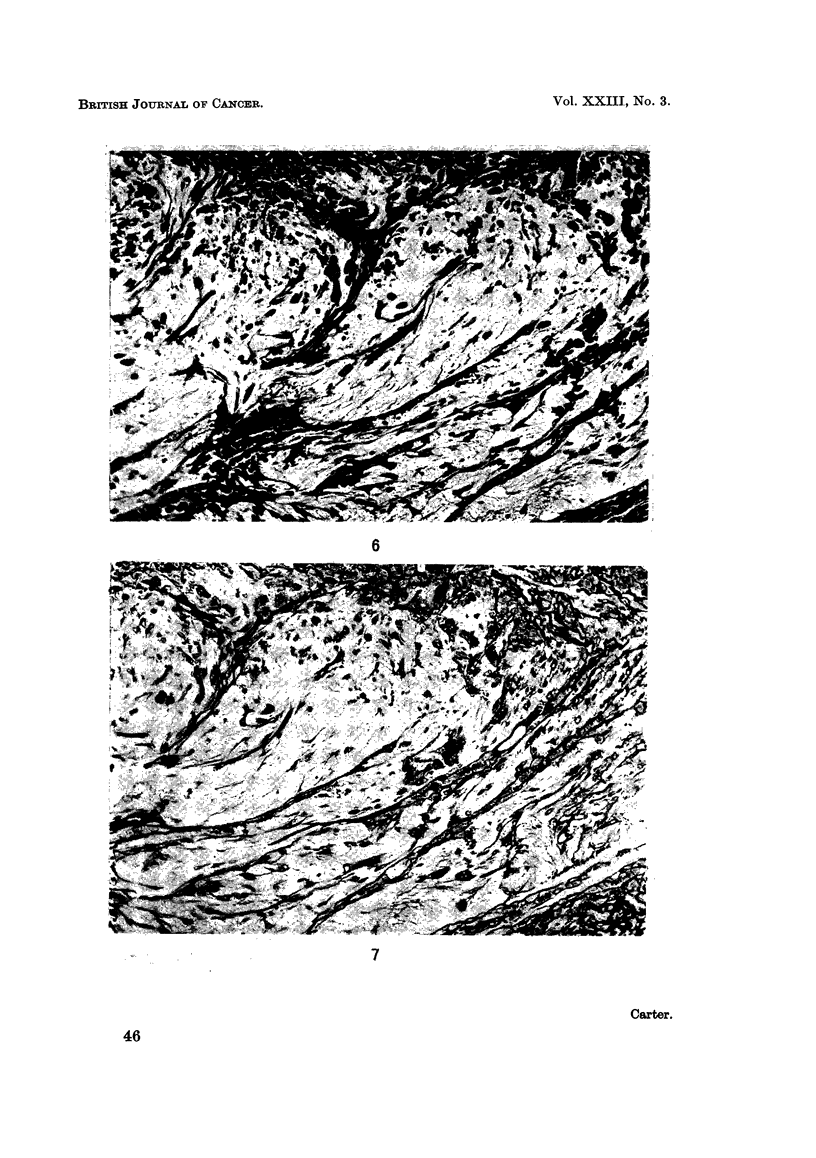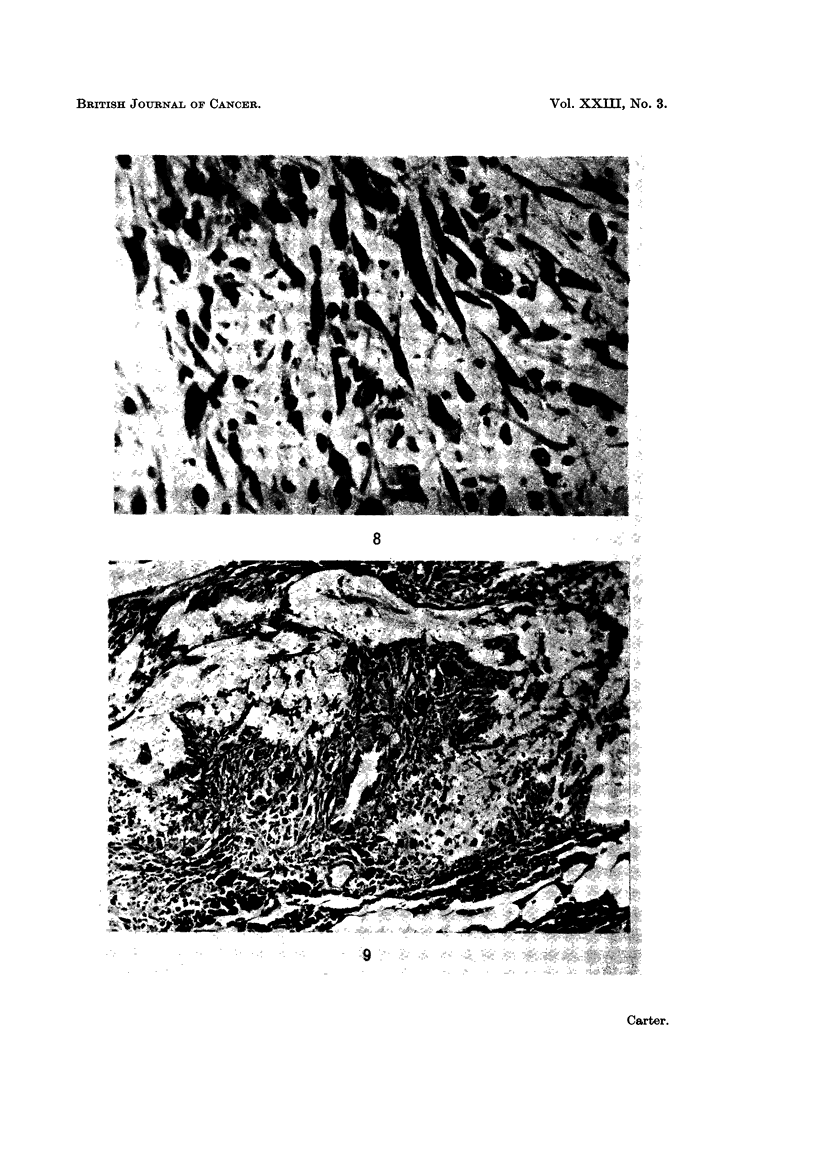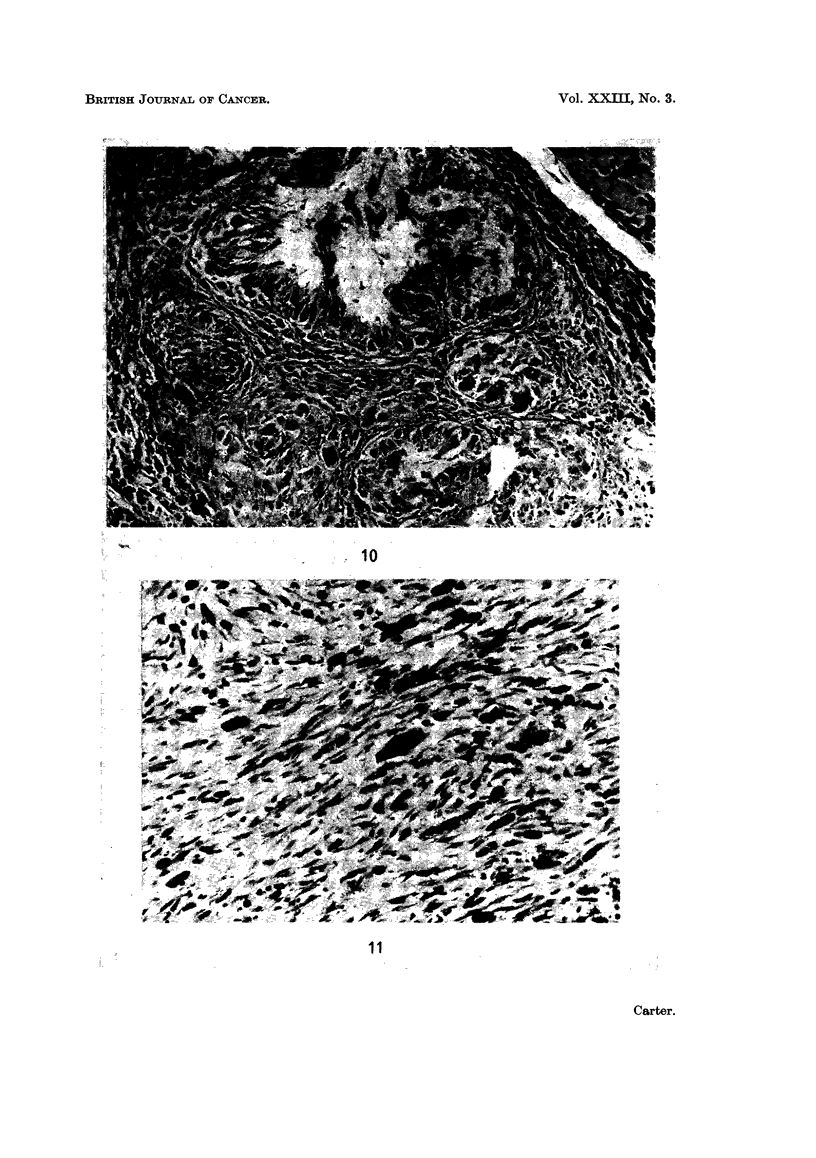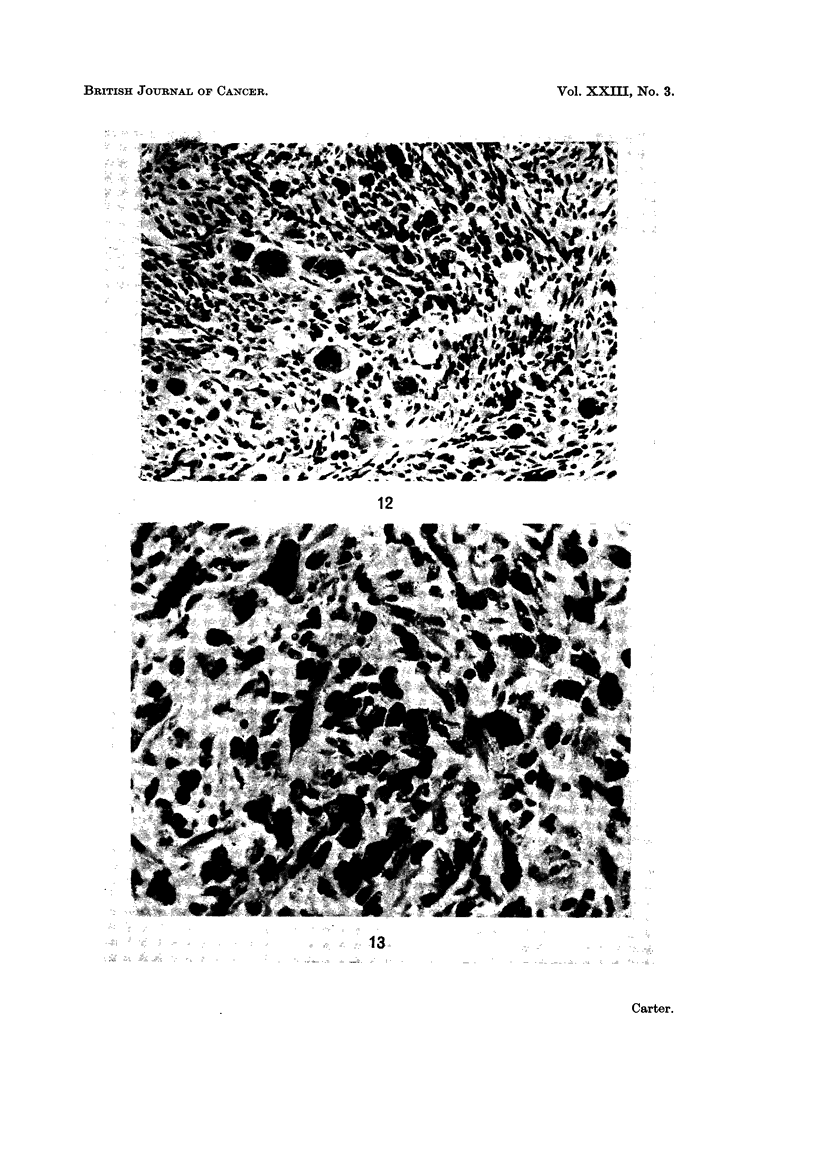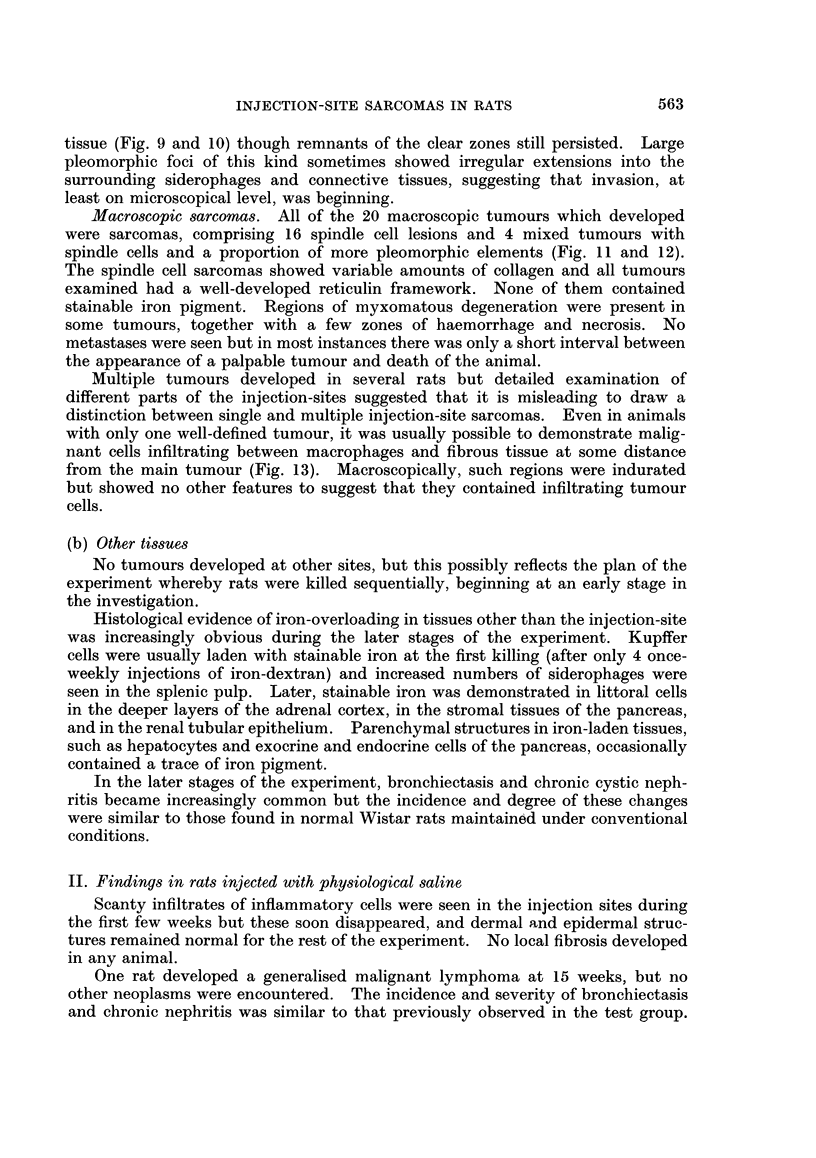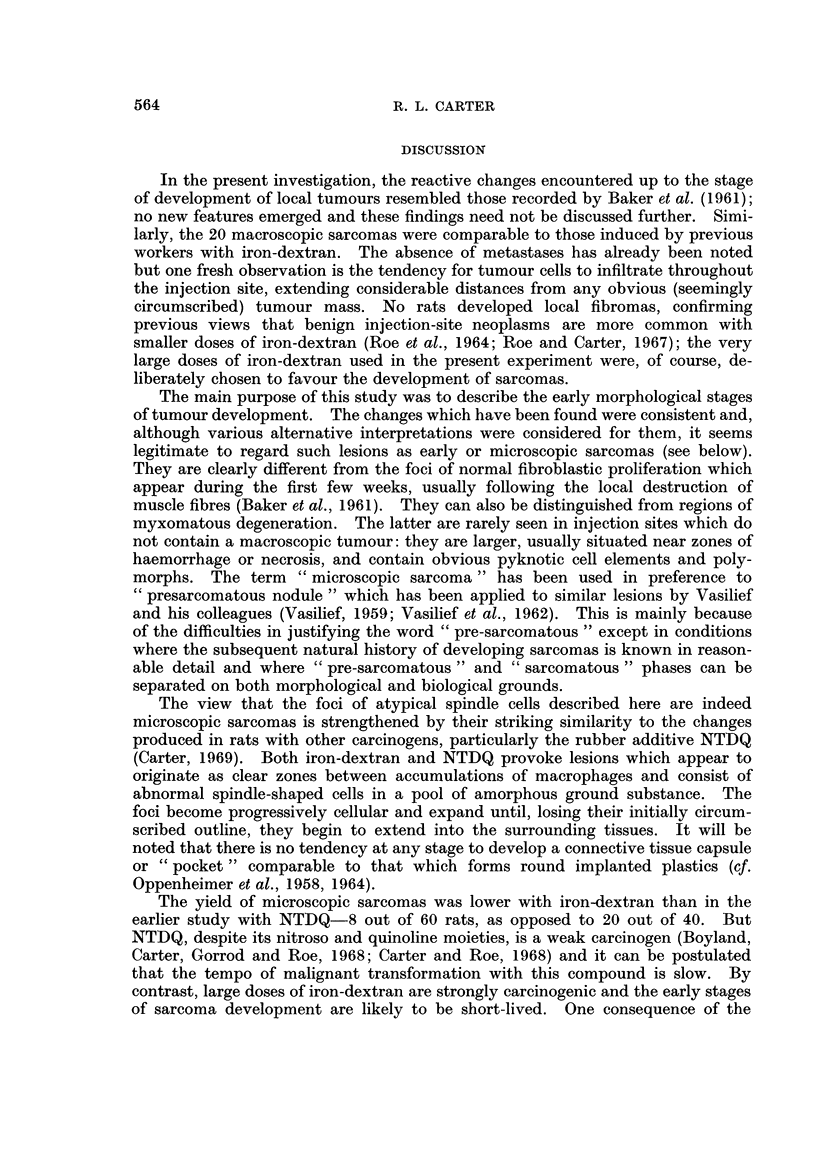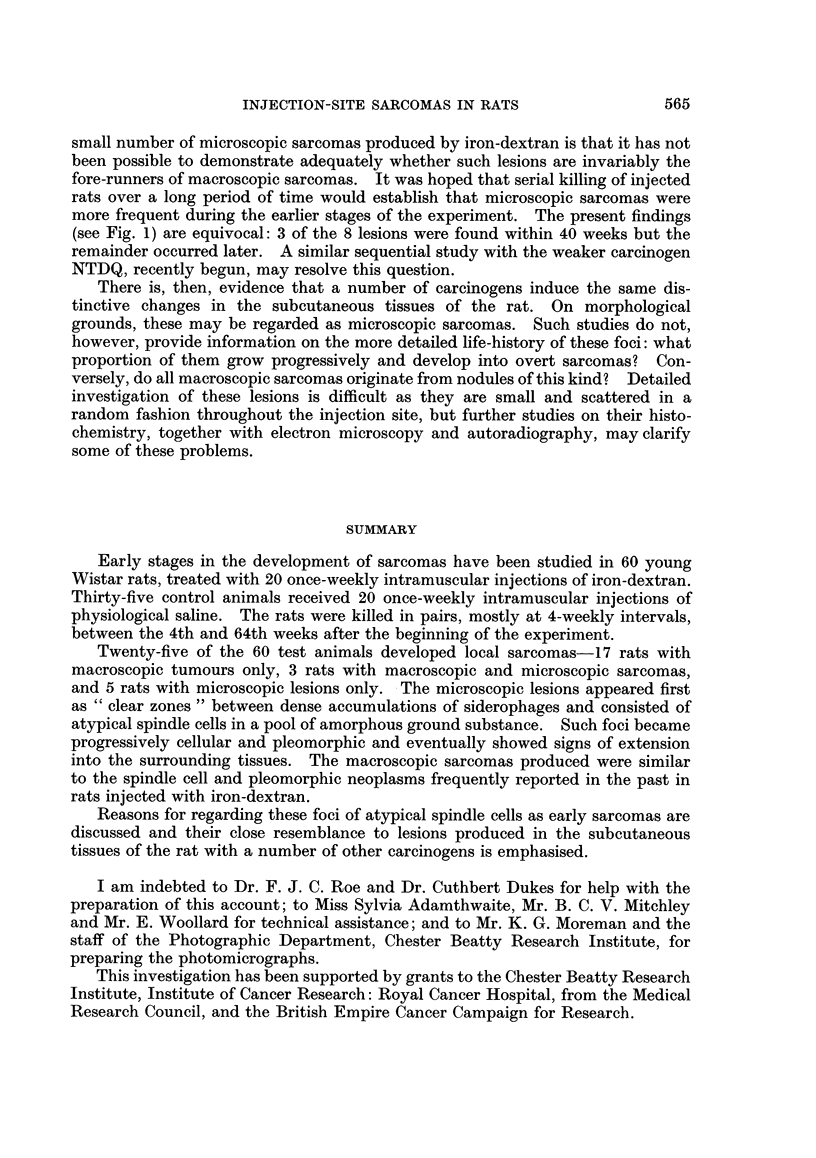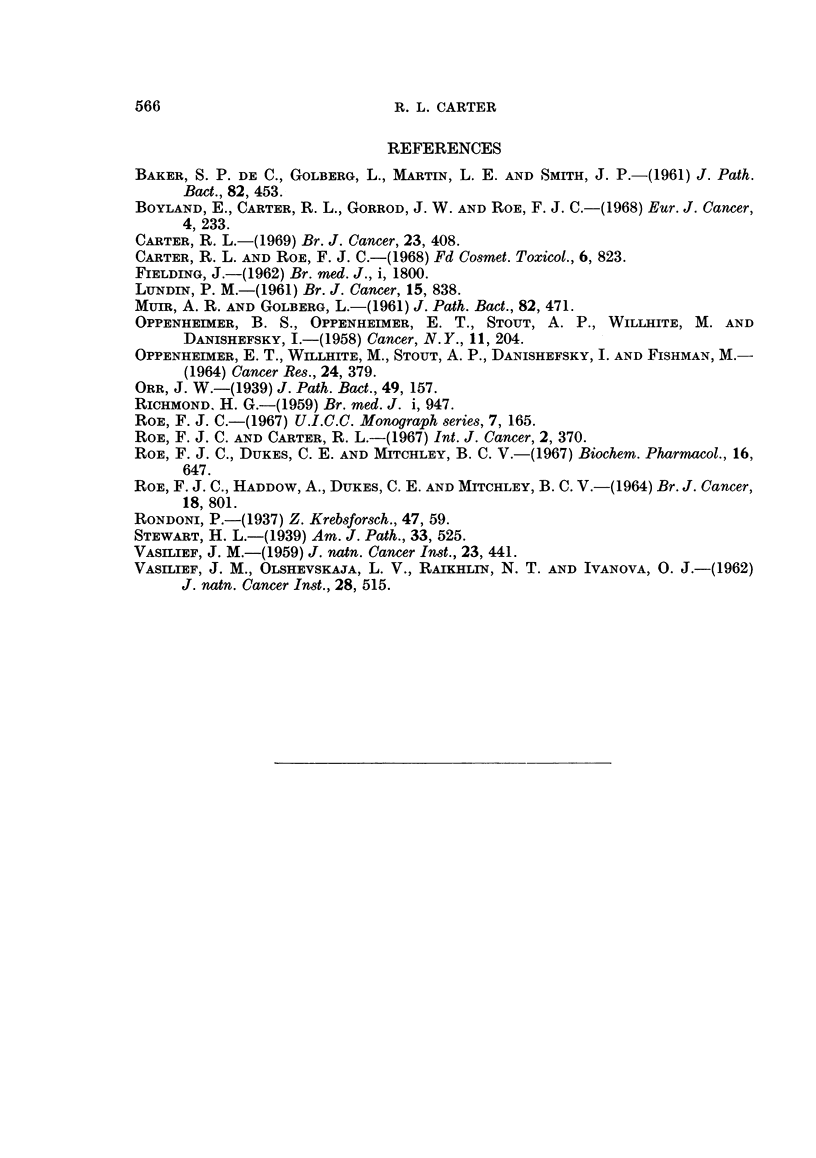# Early development of injection-site sarcomas in rats: a study of tumours induced by iron-dextran.

**DOI:** 10.1038/bjc.1969.69

**Published:** 1969-09

**Authors:** R. L. Carter

## Abstract

**Images:**


### EARLY DEVELOPMENT OF INJECTION-SITE SARCOMAS IN RATS: A STUDY OF TUMOURS INDUCED BY IRON-DEXTRAN

R. L. CARTER

*From the Chester Beatty Research Institute, Institute of Cancer Research: Royal Cancer Hospital, London, S.W.3*

Received for publication April 17, 1969

IN 1959, Richmond described the carcinogenic effect of iron-dextran, injected intramuscularly into rats. Since that time, there have been many accounts of tumour induction by macromolecular iron-complexes in various experimental animals (see Roe, 1967) and the neoplasms produced—predominantly sarcomas—have often been described and illustrated. Much less attention has been paid to the early stages of sarcoma development although several authors allude briefly to changes which they have regarded as preneoplastic or neoplastic in character.

In his original account, Richmond noted that " occasional histiocytes develop enlargement and hyperchromatism of the nucleus associated with mitotic activity and other aberrant changes " and implied that injection-site sarcomas developed from histiocytes—a view which has not been generally accepted. Muir and Golberg (1961) described pale zones, visible to the naked eye, in which some of the earliest stages of tumour development could be identified and similar abnormal foci in the connective tissues were recorded by Lundin (1961) and Fielding (1962). Roe, Haddow, Dukes and Mitchley (1964) described " sarcomata of microscopic size " in injection sites from rats treated with iron-dextran, though they were properly cautious in their evaluation of such lesions, and further examples were briefly described by Roe and Carter (1967).

These changes are of more than academic interest as they occur in the subcutaneous tissues of rats treated with a variety of carcinogens. Similar lesions have been observed in rats injected with the rubber additive polymerised N-nitroso-2,2,4-trimethyl-1,2,dihydroquinoline (NTDQ) (Carter, 1969) and in animals bearing subcutaneous implants of plastic sponges (Roe, Dukes and Mitchley, 1967); it is also probable that the early neoplastic changes induced by implanted pellets of polycyclic hydrocarbons such as 3,4-benzopyrene, 3-methylcholanthrene and 7,12-dimethylbenz(*a*)anthracene provide further examples of these lesions (Rondoni, 1937; Orr, 1939; Stewart, 1939; also Vasilief, 1959; Vasilief *et al.*, 1962). In the present paper, the early malignant changes induced by iron-dextran in the rat are considered in detail.

### MATERIALS AND METHODS

Ninety-five male CB Wistar rats, 5–8 weeks old, were divided into a test group of 60 animals and a control group of 35 animals. The rats were housed in metal cages, 6 animals in each, and maintained on a cubed diet (Dixon and Sons, Ware, Herts.) and water *ad libitum*.

Iron-dextran (" imferon ": Fison's Pharmaceuticals Ltd., Holmes Chapel, Cheshire) Batch No. 376/5 was used. 1 ml. of this preparation contains 50 mg. iron.

Rats in the test group received once-weekly injections of 1 ml. iron-dextran for 20 consecutive weeks, given intramuscularly into the right flank. The total dose of iron administered to each rat was 1 g. The 35 rats in the control group received 20 once-weekly intramuscular injections of 1 ml. physiological saline. During and after their course of injections, all animals were regularly examined and the injection-site carefully palpated.

It was originally intended that the rats, which were strictly randomised within the two groups at the beginning of the experiment, should be killed in predetermined pairs at 4-weekly intervals for a period of 72 weeks. Although satisfactory

FIG. 1.—Development of injection-site sarcomas in rats treated with iron-dextran.

in the early stages of the investigation, this scheme was eventually modified as increasing numbers of the long-term survivors developed rapidly growing tumours at the site of injection and had to be killed prematurely. The experiment was therefore terminated at 64 weeks. The eventual times at which test and control animals were killed are shown in Fig. 1.

Rats were killed with ether and full post-mortem examinations were made. Injection sites from all test and control animals were excised and blocks of tissue were removed from each end and from the middle of the injected region, together with the liver, spleen, kidneys, pancreas, adrenal glands, lungs and any other tissues showing macroscopic abnormalities. This material was fixed in Bouin's solution and 5 $\mu$ paraffin sections were prepared and stained with haematoxylin and eosin. Additional sections of the injection sites were stained with orcein and Van Gieson, Masson's trichrome, Gordon and Sweets' silver impregnation method for reticulin fibres, Perl's method for iron, periodic acid-Schiff (PAS), toluidine blue and Lison's alcian blue and chlorantine fast red.

### RESULTS

It is clearly meaningless to analyse survival in an experiment where the animals were, for the most part, killed at pre-determined intervals; but the times at which rats in the test and control groups were killed are summarised in Fig. 1. The large number of local neoplasms which developed towards the end of the investigation vitiated the original plan for animals to be killed sequentially over a longer span of time.

Tumours at the injection site were confined to rats which were treated with iron-dextran. Twenty-five out of 60 test animals (41·7%) developed local sarcomas—17 rats with macroscopic lesions only, 3 rats with macroscopic and microscopic lesions, and 5 rats with microscopic lesions only. The first macroscopic sarcoma was recognised 31 weeks after the beginning of the experiment. Once palpable through the indurated flank, the tumours grew rapidly and, in most instances, the tumour-bearing rats had to be killed in the ensuing 20 to 30 days. The average time of induction of the macroscopic sarcomas was 45 weeks. The first microscopic sarcoma was seen at 33 weeks; although 3 of these lesions occurred within the first 40 weeks, 5 were not encountered until later.

### *Histopathological Changes*

I. *Findings in rats injected with iron-dextran*

(a) *Injection sites*

As several aspects of the developing tissue reaction to iron-dextran in rats have already been described, notably by Baker, Golberg, Martin and Smith (1961), the following account will deal mainly with those changes which are directly associated with the early development of injection-site tumours. But in order to set these findings in context, some comments will be made on the preceding reactive changes induced by iron-dextran and also on the established sarcomas.

*Non-neoplastic changes.* Injection sites from rats killed after only 4 once-weekly injections of iron-dextran already contained large numbers of macrophages which were heavily loaded with iron. Dermal blood vessels and nerves were surrounded by siderophages and stainable iron often extended inside nerve bundles, presumably taken up by Schwann cells. There were negligible amounts of free extracellular iron. Moderate oedema was present but acute or chronic inflammatory cells were rarely seen. As the number of injections increased, the siderophages formed dense linear accumulations (Fig. 2), a striking feature which was well developed by 8 weeks. By this time there was considerable destruction of muscle fibres, first in the panniculus carnosus, and later in the deeper layers of the body wall. No evidence of regeneration was seen and, as the muscles were destroyed, there was an increasing amount of local fibrosis. Dense fibrous tissue developed which tended to split the previously homogenous mass of siderophages into smaller " islands " of cells (Fig. 3).

Apart from the very ealiest stages, therefore, the histological changes at the injection site involve two principal elements—siderophages and fibrous tissue—although the proportion of each tends to vary in different parts of the same injection site at any one time. It is against this background that neoplastic changes develop.

*Early or microscopic sarcomas.* Nodules of atypical cells were seen in 8 rats and were regarded as microscopic sarcomas. The first indication of impending neoplastic change was the appearance of a " clear zone " between the previously compact masses of siderophages and connective tissue. Such regions, too small to recognise in gross specimens, were sharply demarcated and consisted of a few atypical spindle-shaped cells in a mass of amorphous ground substance (Fig. 4). Some of the cells were multi-nucleate and some in mitosis (Fig. 5). Their cytoplasm was PAS-positive and stained weakly with alcian blue; there was no intracellular iron-pigment. The faintly eosinophilic ground substance was invariably PAS-negative and, in most instances, showed weak metachromasia with toluidine blue and some staining with alcian blue.

There was no limiting fibrous capsule but the connective tissues in and around the foci of atypical cells showed striking changes (Fig. 6 and 7). The surrounding structures contained a dense fibrous stroma but, in the region of the clear zones, collagen and reticulin fibres were greatly reduced in amount and were fragmented; fine fibres predominated but some fibres were unusually thick and coarse in appearance. There was no morphological evidence of collagen formation by the atypical cells in the nodules.

In some regions, the foci of abnormal cells were larger and appeared unequivocally neoplastic. The cells were increased in number and commonly showed two patterns of growth. In some injection sites, the proliferating cells remained isolated and showed no tendency to become organised into a discrete lesion (Fig. 8); in others, the tumour cells formed an almost solid core of neoplastic

---

EXPLANATION OF PLATES

FIG. 2.—Injection site: 8 weeks. Dense accumulation of siderophages showing characteristic orientation in long parallel lines. H. and E. × 120.

FIG. 3.—Injection site: 16 weeks. Siderophages interspersed with dense fibrous tissue. H. and E. × 120.

FIG. 4.—Injection site: 37 weeks. Low-power view of a " clear zone " composed of a few atypical spindle cells and ground substance, surrounded by siderophages and fibrous tissue. PAS. × 120.

FIG. 5.—Injection site: 40 weeks. Clear zone containing a number of pleomorphic spindle cells, some of them in mitosis. PAS. × 120.

FIG. 6.—Injection site: 46 weeks. Disruption and distortion of collagen fibres in a clear zone. The surrounding stroma and siderophages can be seen but there is no connective tissue capsule. Haematoxylin and Van Gieson. × 225.

FIG. 7.—Injection site; 46 weeks. Same injection site showing distortion of reticulin fibres. Silver impregnation (Gordon and Sweets). × 225.

FIG. 8.—Injection site; 46 weeks. Focal proliferation of spindle cells with no organisation into a discrete " tumour ". H. and E. × 357.

FIG. 9.—Injection site: 50 weeks. Well-established microscopic sarcoma composed of clumps of pleomorphic cells (*cf.* Fig. 5). PAS × 120.

FIG. 10.—Injection site: 50 weeks. An almost solid nodule of microscopic sarcoma which is beginning to infiltrate between the adjacent siderophages. PAS × 120.

FIG. 11.—Injection site: 44 weeks. Typical spindle cell sarcoma, interspersed with a few siderophages. H. and E. × 190.

FIG. 12.—Injection site: 55 weeks. A more anaplastic tumour comprised of spindle cell and pleomorphic elements. H. and E. × 225.

FIG. 13.—Injection site: 44 weeks. Malignant cells infiltrating between siderophages some distance from the main tumour mass. H. and E. × 357.

2

3

Carter.

4

5

6

7

Carter.

8

9

Carter.

10

11

Carter.

12

13

tissue (Fig. 9 and 10) though remnants of the clear zones still persisted. Large pleomorphic foci of this kind sometimes showed irregular extensions into the surrounding siderophages and connective tissues, suggesting that invasion, at least on microscopical level, was beginning.

*Macroscopic sarcomas.* All of the 20 macroscopic tumours which developed were sarcomas, comprising 16 spindle cell lesions and 4 mixed tumours with spindle cells and a proportion of more pleomorphic elements (Fig. 11 and 12). The spindle cell sarcomas showed variable amounts of collagen and all tumours examined had a well-developed reticulin framework. None of them contained stainable iron pigment. Regions of myxomatous degeneration were present in some tumours, together with a few zones of haemorrhage and necrosis. No metastases were seen but in most instances there was only a short interval between the appearance of a palpable tumour and death of the animal.

Multiple tumours developed in several rats but detailed examination of different parts of the injection-sites suggested that it is misleading to draw a distinction between single and multiple injection-site sarcomas. Even in animals with only one well-defined tumour, it was usually possible to demonstrate malignant cells infiltrating between macrophages and fibrous tissue at some distance from the main tumour (Fig. 13). Macroscopically, such regions were indurated but showed no other features to suggest that they contained infiltrating tumour cells.

(b) *Other tissues*

No tumours developed at other sites, but this possibly reflects the plan of the experiment whereby rats were killed sequentially, beginning at an early stage in the investigation.

Histological evidence of iron-overloading in tissues other than the injection-site was increasingly obvious during the later stages of the experiment. Kupffer cells were usually laden with stainable iron at the first killing (after only 4 once-weekly injections of iron-dextran) and increased numbers of siderophages were seen in the splenic pulp. Later, stainable iron was demonstrated in littoral cells in the deeper layers of the adrenal cortex, in the stromal tissues of the pancreas, and in the renal tubular epithelium. Parenchymal structures in iron-laden tissues, such as hepatocytes and exocrine and endocrine cells of the pancreas, occasionally contained a trace of iron pigment.

In the later stages of the experiment, bronchiectasis and chronic cystic nephritis became increasingly common but the incidence and degree of these changes were similar to those found in normal Wistar rats maintained under conventional conditions.

II. *Findings in rats injected with physiological saline*

Scanty infiltrates of inflammatory cells were seen in the injection sites during the first few weeks but these soon disappeared, and dermal and epidermal structures remained normal for the rest of the experiment. No local fibrosis developed in any animal.

One rat developed a generalised malignant lymphoma at 15 weeks, but no other neoplasms were encountered. The incidence and severity of bronchiectasis and chronic nephritis was similar to that previously observed in the test group.

### DISCUSSION

In the present investigation, the reactive changes encountered up to the stage of development of local tumours resembled those recorded by Baker *et al.* (1961); no new features emerged and these findings need not be discussed further. Similarly, the 20 macroscopic sarcomas were comparable to those induced by previous workers with iron-dextran. The absence of metastases has already been noted but one fresh observation is the tendency for tumour cells to infiltrate throughout the injection site, extending considerable distances from any obvious (seemingly circumscribed) tumour mass. No rats developed local fibromas, confirming previous views that benign injection-site neoplasms are more common with smaller doses of iron-dextran (Roe *et al.*, 1964; Roe and Carter, 1967); the very large doses of iron-dextran used in the present experiment were, of course, deliberately chosen to favour the development of sarcomas.

The main purpose of this study was to describe the early morphological stages of tumour development. The changes which have been found were consistent and, although various alternative interpretations were considered for them, it seems legitimate to regard such lesions as early or microscopic sarcomas (see below). They are clearly different from the foci of normal fibroblastic proliferation which appear during the first few weeks, usually following the local destruction of muscle fibres (Baker *et al.*, 1961). They can also be distinguished from regions of myxomatous degeneration. The latter are rarely seen in injection sites which do not contain a macroscopic tumour: they are larger, usually situated near zones of haemorrhage or necrosis, and contain obvious pyknotic cell elements and polymorphs. The term " microscopic sarcoma " has been used in preference to " presarcomatous nodule " which has been applied to similar lesions by Vasilief and his colleagues (Vasilief, 1959; Vasilief *et al.*, 1962). This is mainly because of the difficulties in justifying the word " pre-sarcomatous " except in conditions where the subsequent natural history of developing sarcomas is known in reasonable detail and where " pre-sarcomatous " and " sarcomatous " phases can be separated on both morphological and biological grounds.

The view that the foci of atypical spindle cells described here are indeed microscopic sarcomas is strengthened by their striking similarity to the changes produced in rats with other carcinogens, particularly the rubber additive NTDQ (Carter, 1969). Both iron-dextran and NTDQ provoke lesions which appear to originate as clear zones between accumulations of macrophages and consist of abnormal spindle-shaped cells in a pool of amorphous ground substance. The foci become progressively cellular and expand until, losing their initially circumscribed outline, they begin to extend into the surrounding tissues. It will be noted that there is no tendency at any stage to develop a connective tissue capsule or " pocket " comparable to that which forms round implanted plastics (*cf.* Oppenheimer *et al.*, 1958, 1964).

The yield of microscopic sarcomas was lower with iron-dextran than in the earlier study with NTDQ—8 out of 60 rats, as opposed to 20 out of 40. But NTDQ, despite its nitroso and quinoline moieties, is a weak carcinogen (Boyland, Carter, Gorrod and Roe, 1968; Carter and Roe, 1968) and it can be postulated that the tempo of malignant transformation with this compound is slow. By contrast, large doses of iron-dextran are strongly carcinogenic and the early stages of sarcoma development are likely to be short-lived. One consequence of the small number of microscopic sarcomas produced by iron-dextran is that it has not been possible to demonstrate adequately whether such lesions are invariably the fore-runners of macroscopic sarcomas. It was hoped that serial killing of injected rats over a long period of time would establish that microscopic sarcomas were more frequent during the earlier stages of the experiment. The present findings (see Fig. 1) are equivocal: 3 of the 8 lesions were found within 40 weeks but the remainder occurred later. A similar sequential study with the weaker carcinogen NTDQ, recently begun, may resolve this question.

There is, then, evidence that a number of carcinogens induce the same distinctive changes in the subcutaneous tissues of the rat. On morphological grounds, these may be regarded as microscopic sarcomas. Such studies do not, however, provide information on the more detailed life-history of these foci: what proportion of them grow progressively and develop into overt sarcomas? Conversely, do all macroscopic sarcomas originate from nodules of this kind? Detailed investigation of these lesions is difficult as they are small and scattered in a random fashion throughout the injection site, but further studies on their histochemistry, together with electron microscopy and autoradiography, may clarify some of these problems.

### SUMMARY

Early stages in the development of sarcomas have been studied in 60 young Wistar rats, treated with 20 once-weekly intramuscular injections of iron-dextran. Thirty-five control animals received 20 once-weekly intramuscular injections of physiological saline. The rats were killed in pairs, mostly at 4-weekly intervals, between the 4th and 64th weeks after the beginning of the experiment.

Twenty-five of the 60 test animals developed local sarcomas—17 rats with macroscopic tumours only, 3 rats with macroscopic and microscopic sarcomas, and 5 rats with microscopic lesions only. The microscopic lesions appeared first as " clear zones " between dense accumulations of siderophages and consisted of atypical spindle cells in a pool of amorphous ground substance. Such foci became progressively cellular and pleomorphic and eventually showed signs of extension into the surrounding tissues. The macroscopic sarcomas produced were similar to the spindle cell and pleomorphic neoplasms frequently reported in the past in rats injected with iron-dextran.

Reasons for regarding these foci of atypical spindle cells as early sarcomas are discussed and their close resemblance to lesions produced in the subcutaneous tissues of the rat with a number of other carcinogens is emphasised.

I am indebted to Dr. F. J. C. Roe and Dr. Cuthbert Dukes for help with the preparation of this account; to Miss Sylvia Adamthwaite, Mr. B. C. V. Mitchley and Mr. E. Woollard for technical assistance; and to Mr. K. G. Moreman and the staff of the Photographic Department, Chester Beatty Research Institute, for preparing the photomicrographs.

This investigation has been supported by grants to the Chester Beatty Research Institute, Institute of Cancer Research: Royal Cancer Hospital, from the Medical Research Council, and the British Empire Cancer Campaign for Research.

### REFERENCES

BAKER, S. P. DE C., GOLBERG, L., MARTIN, L. E. AND SMITH, J. P.—(1961) *J. Path. Bact.*, **82**, 453.
BOYLAND, E., CARTER, R. L., GORROD, J. W. AND ROE, F. J. C.—(1968) *Eur. J. Cancer*, **4**, 233.
CARTER, R. L.—(1969) *Br. J. Cancer*, **23**, 408.
CARTER, R. L. AND ROE, F. J. C.—(1968) *Fd Cosmet. Toxicol.*, **6**, 823.
FIELDING, J.—(1962) *Br. med. J.*, i, 1800.
LUNDIN, P. M.—(1961) *Br. J. Cancer*, **15**, 838.
MUIR, A. R. AND GOLBERG, L.—(1961) *J. Path. Bact.*, **82**, 471.
OPPENHEIMER, B. S., OPPENHEIMER, E. T., STOUT, A. P., WILLHITE, M. AND DANISHEFSKY, I.—(1958) *Cancer, N.Y.*, **11**, 204.
OPPENHEIMER, E. T., WILLHITE, M., STOUT, A. P., DANISHEFSKY, I. AND FISHMAN, M.—(1964) *Cancer Res.*, **24**, 379.
ORR, J. W.—(1939) *J. Path. Bact.*, **49**, 157.
RICHMOND, H. G.—(1959) *Br. med. J.* i, 947.
ROE, F. J. C.—(1967) *U.I.C.C. Monograph series*, **7**, 165.
ROE, F. J. C. AND CARTER, R. L.—(1967) *Int. J. Cancer*, **2**, 370.
ROE, F. J. C., DUKES, C. E. AND MITCHLEY, B. C. V.—(1967) *Biochem. Pharmacol.*, **16**, 647.
ROE, F. J. C., HADDOW, A., DUKES, C. E. AND MITCHLEY, B. C. V.—(1964) *Br. J. Cancer*, **18**, 801.
RONDONI, P.—(1937) *Z. Krebsforsch.*, **47**, 59.
STEWART, H. L.—(1939) *Am. J. Path.*, **33**, 525.
VASILIEF, J. M.—(1959) *J. natn. Cancer Inst.*, **23**, 441.
VASILIEF, J. M., OLSHEVSKAJA, L. V., RAIKHLIN, N. T. AND IVANOVA, O. J.—(1962) *J. natn. Cancer Inst.*, **28**, 515.